# Molecular Diagnosis of Drug-Resistant Tuberculosis; A Literature Review

**DOI:** 10.3389/fmicb.2019.00794

**Published:** 2019-04-16

**Authors:** Thi Ngoc Anh Nguyen, Véronique Anton-Le Berre, Anne-Laure Bañuls, Thi Van Anh Nguyen

**Affiliations:** ^1^UMR MIVEGEC, Institute of Research for Development, Centre National de la Recherche Scientifique, University of Montpellier, Montpellier, France; ^2^Laboratory of Tuberculosis, Department of Bacteriology, National Institute of Hygiene and Epidemiology, Hanoi, Vietnam; ^3^LMI Drug Resistance in South East Asia, National Institute of Hygiene and Epidemiology, Hanoi, Vietnam; ^4^LISBP, CNRS, INRA, INSA, Université de Toulouse, Toulouse, France

**Keywords:** tuberculosis, drug resistance, diagnosis, mutations, molecular markers, challenges

## Abstract

Drug-resistant tuberculosis is a global health problem that hinders the progress of tuberculosis eradication programs. Accurate and early detection of drug-resistant tuberculosis is essential for effective patient care, for preventing tuberculosis spread, and for limiting the development of drug-resistant strains. Culture-based drug susceptibility tests are the gold standard method for the detection of drug-resistant tuberculosis, but they are time-consuming and technically challenging, especially in low- and middle-income countries. Nowadays, different nucleic acid-based assays that detect gene mutations associated with resistance to drugs used to treat tuberculosis are available. These tests vary in type and number of targets and in sensitivity and specificity. In this review, we will describe the available molecular tests for drug-resistant tuberculosis detection and discuss their advantages and limitations.

## Introduction

Drug resistance is a major challenge for tuberculosis (TB) treatment and eradication. The number of drug-resistant TB (DR-TB) cases is increasing worldwide: the estimated number of new cases of multidrug-resistant TB (MDR-TB, defined as TB resistant at least to isoniazid and rifampicin) or rifampicin-resistant TB (RR-TB) was 600,000 in 2016 compared with 480,000 in 2014 (WHO, 2017a,b). The treatment of MDR-TB is complex, much more expensive than the treatment of non-MDR-TB, and is associated with important side effects for the patients. The treatment success rate decreases from 83% for patients with newly diagnosed or relapse non-DR-TB who start treatment with a first-line regimen (2015 cohort) to 54% for MDR/RR-TB (2014 cohort) and to 30% for extensively drug-resistant TB (XDR-TB, defined as MDR-TB showing resistance also to at least one fluoroquinolone and one second-line injectable agent; 2014 cohort) (WHO, 2017b). In 2015, only one–third of new patients with bacteriologically confirmed TB and previously treated TB patients underwent drug susceptibility testing (DST) for rifampicin (RIF). The others were treated based only on smear-positive results without DST and differentiation of *Mycobacterium tuberculosis* (MTB) from non-tuberculous mycobacteria (NTM). In low and middle income countries, DST accessibility is limited by its high cost and complexity and by technical constraints (maintenance and environmental conditions). Such difficulties could result in the transmission and emergence of highly DR-MTB strains due to inappropriate treatments.

Drug susceptibility testing, the reference method for DR-TB detection, is based on the proportional agar method on Lowenstein-Jensen medium (Canetti et al., 1969). As this culture method is time-consuming (up to 3–8 weeks to get results), other solid culture methods, such as Middlebrook agar, have been developed to reduce the turn-around time to 10–12 days (Brady et al., 2008; Nguyen et al., 2015). Liquid culture methods have been used for faster detection with high sensitivity (Lawson et al., 2013). For instance, the BACTEC Mycobacteria Growth Indicator Tube (MGIT^TM^) are barcoded for the automated processing of large numbers of samples (up to 960 tubes)^1^ with a turn-around time between 10 and 30 days (Lawson et al., 2013). However, liquid culture methods come with several disadvantages: invisible contamination, overgrowth of NTM, and need of expensive complex systems (Singhal et al., 2012; Lawson et al., 2013; Nguyen et al., 2015). Moreover, culture-based methods generally require well-trained personnel and high biosafety-level laboratories for the protection of the technicians and the environment. Thus, the use of culture methods for DR-TB detection is limited, especially in low- or middle-income countries.

Previous studies demonstrated that in MTB strains, drug resistance is mainly acquired through spontaneous mutations, especially single nucleotide polymorphisms (SNPs), in the circular chromosome (McGrath et al., 2014). For each anti-TB drug, mutations in one or several genes have been described, and each mutation relates to different levels of drug resistance. Mutation frequency also can be variable. For instance, 97% of the cases of resistance to RIF are linked to mutations in the *rpo*B gene, mostly in an 81 bp hotspot region (codon 507 to codon 533; *Escherichia coli* numbering system, used throughout the text for RIF) (Laurenzo and Mousa, 2011). Mutations at codons 526–531 of *rpoB* show the highest frequency and confer high-level RIF resistance. Isoniazid (INH) resistance is acquired through mutations in the *kat*G, *inh*A and its promoter, *ahp*C, *ndh*, and *fur*A genes but mainly in *kat*G, *inh*A and its promoter (Laurenzo and Mousa, 2011). The most frequent *kat*G mutations (50–90%) are found at codon 315 and confer high-level resistance to INH. In ethambutol (EMB)-resistant isolates, mutations at codon 306 of the *emb*B gene are the most frequent (47–62%). Mutations associated with resistance to second-line drugs also have been described. In streptomycin (STR)-resistant MTB isolates, the most frequent mutations are found at codons 43 and 88 of the *rps*L gene, and codon 514 of the *rrs* gene (Laurenzo and Mousa, 2011; Nguyen H.Q. et al., 2017). Resistance to fluoroquinolones (FQs) arises through mutations in the *gyr*A and *gyr*B genes. About 60–70% of quinolone-resistant MTB isolates harbor mutations in the quinolone resistance-determining region of *gyr*A, with the highest frequency at codon 94, followed by codon 90, 91, and 88 (Laurenzo and Mousa, 2011). Mutations in the *gyr*B gene are rare. Resistance to second-line injectable drugs, including amikacin (AMK), kanamycin (KAN), and capreomycin (CAP), is mainly associated with *rrs* gene mutations. Approximately 70–80% of CAP resistance and about 60% of KAN resistance are caused by the *rrs* A1401G mutation. Although the mechanisms of drug resistance is not clear in about 10–40% of DR-MTB isolates without mutations, the detection of known mutations enables to identify a high proportion of DR-MTB (Jeanes and O’Grady, 2016).

Therefore, many nucleic acid-based assays that detect mutations associated with anti-TB drug resistance have been developed recently, in order to provide affordable, accurate, simple, and rapid diagnostic tests for DR-TB detection. In this review, we will describe the commercially available molecular tests for DR-TB detection and discuss their advantages and limitations (see Supplementary Table S1 for a summary of the available molecular tests).

## Molecular Assays for the Detection of Drug Resistance in MTB

### DNA Line Probe Assays

Line probe assays (LPAs) are basically DNA–DNA hybridization assays that allow the simultaneous detection of different mutations by using multiple probes^2^ (Makinen et al., 2006). After DNA extraction and target amplification, amplicons are hybridized to specific oligonucleotide probes that are complementary to the target sequences and are immobilized on the surface of a strip. After several post-hybridization washes to remove non-specific binding, the amplicon-probe hybrids are visualized by eye as colored bands on the strip. The turnaround time of the whole assay is 5–7 h (Makinen et al., 2006; Mitarai et al., 2012).

Although several LPAs have been developed, most of them focus only on the hotspot regions of drug-resistance and different assays target different genes. For instance, the INNO-LiPA Rif TB LPA (Innogenetics, Zwijndrecht, Belgium) analyzes only the *rpo*B hotspot region (codon 509 to codon 534; Asp516Val, His526Tyr, His526Asp, and Ser531Leu mutations) for MTB identification and RIF resistance screening (WHO, 2008). The AID TB Resistance LPA (Aid Diagnostika GmbH) includes three modules to detect first-line and second-line anti-TB drug resistance in culture and clinical specimens^3^ (Ritter et al., 2014). Module 1 targets *rpo*B, *kat*G, and the *inhA* promoter for RIF and INH resistance screening; module 2 covers *rps*L and *rrs* to detect aminoglycoside resistance (STR, AMK, CAP); and module 3 analyzes *gyr*A and *emb*B for FQ and EMB resistance detection. The three modules include wild type and mutant probes to cover the most common mutations. The AID TB Resistance LPA showed high sensitivity and specificity for the detection of RIF, INH, STR, FQs, and second-line injectable agents resistance (between 90 and 100%), but lower sensitivity for EMB resistance (72.9%) (Molina-Moya et al., 2014). However, with the AID TB Resistance LPA, uninterpretable results have been reported in up to 8.3% of smear-positive and 65% of smear-negative samples (Deggim-Messmer et al., 2016).

Currently, the LPAs recommended by WHO for the initial drug resistance screening of sputum smear-positive samples include GenoType MTBDR*plus*, GenoType MTBDR*sl* (Hain LifeScience GmbH, Germany), and Nipro NTM+MDR-TB (Nipro Co., Osaka, Japan) (WHO, 2017b). GenoType MTBDR*plus* VER2.0 has the advantage of detecting both RIF and INH resistance by screening mutations in *rpo*B, *kat*G, and the *inhA* promoter^4^. GenoType MTBDR*sl* VER1.0 and VER2.0 detect the MTB complex and its resistance to FQs, EMB and aminoglycosides/cyclic peptides by analyzing the *gyr*A, *gyr*B, *rrs*, *emb*B, and *eis* genes^5^. It may be used as initial test for patients with confirmed RR-TB or MDR-TB (WHO, 2017b). Nipro NTM+MDR-TB detects MDR-TB cases by targeting *rpo*B, *kat*G, and *inh*A and also differentiates four important *Mycobacterium* species (MTB, *M. avium*, *M. intracellulare*, and *M. kansasii*) that cause the human disease (Ruesch-Gerdes and Ismail, 2015; Nathavitharana et al., 2016).

GenoType MTBDR*plus* VER2.0 shows good accuracy for the detection of MDR isolates in smear-positive specimens (sensitivity between 83.3 and 96.4%, and specificity between 98.6 and 100%) (Bai et al., 2016; Nathavitharana et al., 2016; Dantas et al., 2017; Meaza et al., 2017). The Nipro NTM+MDRTB strips also show high specificity (between 97 and 100%) for INH and RIF resistance screening in cultured isolates and clinical (sputum) samples, but sensitivity varies between studies (from 50 to 95%) (Mitarai et al., 2012; Ruesch-Gerdes and Ismail, 2015; Nathavitharana et al., 2016). Regarding second-line drugs, GenoType MTBDR*sl* VER2.0 displays high sensitivity and specificity (between 91 and 100%) for detecting FQ resistance, but variable sensitivity and specificity for the screening of resistance to second-line injectable drugs (SLIDs) (Bang et al., 2016; Brossier et al., 2016; Gardee et al., 2017). Therefore, the overall GenoType MTBDR*sl* VER2.0 specificity (between 59 and 100%) and sensitivity (between 83 and 87%) for detecting XDR isolates differ among studies. Moreover, uninterpretable results were reported for drug resistance screening in sputum specimens, especially smear-negative samples (Tomasicchio et al., 2016; Meaza et al., 2017). In addition, when GenoType MTBDR*sl* (both VER1.0 and VER2.0) is used for FQ resistance screening, some synonymous and non-synonymous mutations (i.e., that do not and that do change the encoded amino acid, respectively) in the *gyr*A gene prevent the hybridization of either the wild type or mutant probe, leading to false-resistance results. Although these mutations (T80A + A90G, gcG/gcA A90A, and atC/atT I92I) are not frequent, they account for around 7% of all MDR-TB strains in some studied regions (Ajileye et al., 2017).

In conclusion, LPAs are rapid, simple and easy to perform. Result analysis (manually or automatically) is simple. However, LPAs require complex laboratory infrastructure and expensive equipment that is normally only available in reference laboratories (WHO, 2017c). The number of uninterpretable results is high, and LPA target coverage is limited to the main mutations. Thus, their sensitivity and specificity vary according to the mutation prevalence in the area under study.

### Real-Time PCR Assays

Real-time PCR is now broadly applied for the development of rapid diagnostic tests. Two main approaches are commonly used in real-time PCR: (i) the use of non-specific fluorescent dyes to detect any double-stranded DNA generated by PCR amplification, and (ii) the use of sequence-specific probes tagged with a fluorescent reporter for the specific detection of the hybridization between probes and amplicons^6^. Each probe has a specific melting temperature (Tm), and a Tm change reflects the presence of mutations in the target. This feature has been used to develop real-time PCR tests for drug resistance screening.

Some types of probes can detect mutations conferring drug resistance in MTB, such as dual labeled linear probes (Edwards et al., 2001; Espasa et al., 2005; Liu et al., 2013) and sloppy molecular beacon (SMB) probes (Chakravorty et al., 2011, 2012, 2015; Roh et al., 2015). The dual labeled probes (DLPs) consist of a fluorescence-quencher pair that generates different melting profiles between wild-type and mutant sequences when they hybridize to amplified targets (Roh et al., 2015). Besides, SMB probes consist of stem-loop structures that are thermodynamically more stable than DLPs. SMB probes have the highest sensitivity and specificity (up to 100%) for the detection of mutations in the 81 bp RIF resistance determining region. The presence of 10-fold excess of NTM DNA or 10^5^-fold excess of human DNA does not affect the results with SMB probes (Chakravorty et al., 2012). SMB probes can detect correctly 100% of mutations in 2 pg DNA templates. This suggests that SMB probes can detect *rpo*B gene mutations in both smear-positive and smear-negative samples. SMB probes identify heteroresistance in a mixture of 10% of mutant DNA and 90% of wild type DNA, while dual labeled probes require at least 40% of mutant DNA (Roh et al., 2015). Although the limit of detection depends on the mutation, overall, SMB probes show higher sensitivity than dual labeled probes.

The reduced risk of contamination (all steps after DNA extraction are performed in one tube) and the shorter turn-around time (compared with LPAs because of the absence of the hybridization step) are among the advantages of real-time PCR. However, real-time PCR has some remarkable disadvantages. First, it requires expensive, specific equipment and skilled technicians. Second, the number of probes that can be used in one reaction is limited, resulting in limited target numbers. This is a major drawback for MTB, a pathogen that can harbor many mutations. Third, the size range of the amplified targets should be limited to 75–200 bp for efficiently detecting multiple mutations (see text foot note 6).

The following sections present some examples of real-time PCR-based commercial kits and new strategies for DR-TB detection.

#### Xpert MTB/RIF and Xpert MTB/RIF Ultra

Xpert MTB/RIF (Cepheid, United States), a fast molecular-based test, is endorsed by WHO for the detection of the MTB complex and RIF resistance screening in suspected cases (WHO, 2017b). This test was first recommended in 2010 for the diagnosis of pulmonary TB in adults from sputum specimens. Since 2013, it has been recommended also for the diagnosis of TB in children and of some specific extra-pulmonary forms.

The Xpert MTB/RIF assay uses semi-quantitative nested real-time PCR to amplify a fragment containing the 81 bp hotspot region of the *rpo*B gene (codons 507–533) that is then hybridized to five molecular beacon probes (Bunsow et al., 2014; Steingart et al., 2014; Ochang et al., 2016). Each probe covers a separate sequence and is labeled with a fluorescent dye. The whole experiment is performed in a self-contained cartridge, like a mini-laboratory, to minimize cross-contamination between samples. Sensitivity and specificity for smear-positive samples can reach 100 and 99%, respectively, and for smear-negative samples are 67 and 99%, respectively, compared to the standard culture-based DST. It significantly decreases the detection time of RIF resistance from 4 to 8 weeks (culture and DST) to 2 h. It has immediately a good impact on patients because it allows starting rapidly the MDR-TB treatment. Moreover, Xpert MTB/RIF increases of 23% the MTB detection rate among culture-confirmed cases compared with smear microscopy, with high accuracy of TB detection and limiting the misdiagnosis between MTB and NTM (Steingart et al., 2014; Sharma et al., 2015).

However, some studies reported false-positive results with Xpert MTB/RIF due to silent mutations [e.g., at codon 514 of *rpo*B (Bunsow et al., 2014)], and false-negative results because of the impossibility to detect RIF-resistance mutations outside the hotspot region [e.g., mutations at codon 572 (Sanchez-Padilla et al., 2015)]. Consequently, Xpert MTB/RIF scope might be limited, for instance, in Swaziland where more than 30% of patients with RR-TB carry the I572F mutation (I491F in the original paper, according to the MTB numbering system). In addition, this test does not detect mutations in genes associated with INH resistance, but uses RIF resistance as a proxy for MDR-TB detection (Manson et al., 2017). Consequently, many INH mono-resistant TB cases are misdiagnosed. Recently, whole genome sequencing (WGS) studies discovered that INH resistance arises before RIF resistance in all lineages, geographical regions and time periods. As Xpert MTB/RIF can detect only RIF resistance, it is unable to identify MDR-TB in its earliest form (i.e., INH mono-resistance). In addition, it must always be used together with other tests, such as DST, to confirm and identify the whole resistance phenotype of each MTB isolate. Another remarkable limitation is its high cost due to the use of complex GeneXpert system and disposable cartridges (the GeneXpert apparatus costs between US$12 000 and $71 000, depending on the number of test modules, and the price of each single-use test cartridge is $9⋅98) (WHO, 2017b; Walzl et al., 2018). Therefore, its use as initial diagnostic test for all suspected TB cases is unaffordable in many high TB-burden countries. According to a WHO report (2016), only 15 of the 48 high TB-burden countries have used the Xpert tests for all suspected TB cases (i.e., 10% of all estimated TB cases globally in 2015) (WHO, 2017b). Finally, the GeneXpert machine requires a constant electricity source and is sensitive to heat and dust. A number of machine failures has been reported due to these problems (Walzl et al., 2018).

Recently, the WHO evaluated the next-generation Xpert MTB/RIF Ultra system (Cepheid) that has a larger amplification chamber to increase the amount of sputum and two additional targets (IS1081 and IS6110) to identify MTB (Chakravorty et al., 2017; Perez-Risco et al., 2018). The new Ultra cartridge can be used in the old GeneXpert machine and has the same price as the old one (WHO, 2017d). However, the analytical sensitivity is significantly increased, more than 10 times. Its higher MTB detection sensitivity (16 bacilli/ml compared with 131 bacilli/ml for the current Xpert MTB/RIF cartridge) facilitates MTB screening in specimens with low numbers of bacilli, such as sputum samples from children and from patients co-infected by HIV, and in difficult-to-diagnose cases, such as smear-negative pulmonary and extra-pulmonary TB. As a result of its higher sensitivity, Xpert Ultra specificity for MTB detection is lower than that of Xpert MTB/RIF^7^ (WHO, 2017b). However, RR-TB detection accuracy is similar with both cartridge types. Xpert MTB/RIF Ultra overcomes some limitations of the current cartridge by excluding the silent *rpo*B mutations Q513Q and F514F (Chakravorty et al., 2017). Therefore, in the latest report, WHO recommended to use the Ultra cartridge as initial diagnostic test for all adults and children with signs and symptoms of TB, and also for screening some extra-pulmonary specimens, such as cerebrospinal fluid, lymph node and tissue samples (WHO, 2017c). Interestingly, Xpert XDR cartridge is in development for the detection of XDR-TB.

Finally, a new device named GeneXpert Omni (Cepheid, United States) is under development. It uses the same cartridges as the Xpert system, but will be smaller, lighter and cheaper than the current Xpert system. It will have a 4-h battery, and thus will be better adapted to overcome the requirement of a stable electric supply^8^.

#### Genedrive MTB/RIF ID Kit

The Genedrive MTB/RIF ID Kit (Epistem, United Kingdm) is an innovative system developed after the Xpert test for the detection of MTB and RR-TB from raw sputum samples. It is a portable low-power thermal cycling apparatus (560 g in weight) that can be operated using a 12V DC power supply and used at TB point-of-care sites (Niemz and Boyle, 2012). The system uses a simple paper-based DNA extraction method combined with asymmetric real-time PCR and a proprietary hybridization probe technology (Highlighter Probes) (Castan et al., 2014). The composite paper is chemically treated to decontaminate and extract DNA from bacteria without any additional equipment, such as vortex or centrifuge. The system employs multiplex real-time PCR to target two regions: a short repetitive region (the REP13E12 family), and the 81 bp hotspot region of *rpo*B. The Highlighter Probes detect the most important mutations associated with RIF resistance at codons 516, 526, and 531, with an overall sensitivity for *rpo*B mutation detection of 72.3%. For MTB identification, Genedrive MTB/RIF ID is comparable to the Xpert assay (100% vs. 93.5% of sputum samples). The platform can detect as low as five genome copies. It is user-friendly and fast (results in 75 min). Although the Genedrive system can analyze only eight samples per working day^9^, the low price ($4000 for the system and $10–$17 for the disposable cartridge) makes the assay affordable for low-income regions (Niemz and Boyle, 2012). Due to its light weight, stable power supply and capacity to function without air conditioning (up to 40°C), screening with the Genedrive system could be implemented at many acid fast bacilli (AFB) smear microscopy centers (WHO, 2017c).

#### Anyplex II MTB/MDR and MTB/XDR

The Anyplex kits (Seegene, South Korea) have been designed for the detection of MTB, MDR-TB, and XDR-TB from sputum and bronchial wash samples, culture isolates, and fresh tissues based on a semi-automated multiplex real-time PCR method^10^. The two proprietary technologies use dual-priming oligonucleotides (DPO^TM^) and tagging-oligonucleotide cleavage and extension (TOCE^TM^) and the real-time PCR CFX96^TM^ apparatus for the highly specific detection of specific SNPs. The Anyplex kits can identify multiple targets in one reaction because the PCR CFX96^TM^ system allows the simultaneous detection of five different fluorescent dyes. Specifically, Anyplex II MTB/MDR detects the 34 most frequent mutations in *rpo*B, *kat*G, and *inh*A for MDR-TB screening, and the MTB/XDR kit detects the 13 main mutations in the *gyr*A and *rrs* genes and *eis* promoter associated with XDR-TB (see text foot note 10) (Igarashi et al., 2017). The entire protocol requires 3.5 h from DNA extraction to result interpretation using the provided software (Molina-Moya et al., 2015).

The Anyplex MTB/MDR kit accurately detects more than 83% of MTB complex bacilli in pulmonary and extra-pulmonary samples, and therefore, is much more sensitive than the AFB smear microscopy method (Sali et al., 2016), and can improve the diagnosis of extra-pulmonary TB with high specificity. In evaluation studies of both kits (Molina-Moya et al., 2015; Sali et al., 2016; Igarashi et al., 2017), specificity ranged between 94 and 100% for the detection of MTB resistant to all targeted drugs in clinical specimens, and sensitivity was between 50 and 100%. The lowest sensitivity was obtained for FQ resistance screening, but was similar to that of pyrosequencing and GenoType MTBDR*sl* (Molina-Moya et al., 2015). Anyplex MTB/MDR sensitivity for detecting resistance to RIF was always higher than 90% for all sample types (Molina-Moya et al., 2015; Sali et al., 2016; Igarashi et al., 2017). This real-time PCR method seems to be better than the Xpert technology because of the very low rate of false-positive results. However, the limited number of targets remains a limitation. Overall, the Anyplex II MTB/MDR and MTB/XDR kits are potential tools for the efficient and rapid detection of MTB and DR-TB in clinical specimens, especially for the diagnosis of extra-pulmonary TB.

#### Digital PCR

Heteroresistance is found in 9–20% of clinical isolates, and up to 25.8% of clinical samples in some high TB incidence regions (Zhang X. et al., 2012). Heteroresistance is the result of the super-infection by at least two isolates, or of the evolution of a single isolate leading to various subpopulations of drug-resistant and -susceptible bacteria in the presence of antibiotics (Pholwat et al., 2013). The detection of heteroresistance is still a challenge for rapid DR-TB diagnosis, even when using sequencing methods (Folkvardsen et al., 2013). Pholwat et al., developed an assay based on digital PCR (Pholwat et al., 2013) that can identify and quantify different resistant subpopulations in mixtures containing as little as one XDR-MTB among thousand susceptible MTB bacilli. Digital PCR is basically a quantitative PCR that relies on the partitioning of samples into thousands of individual small-volume reactions before amplification (Morley, 2014). In these compartments, the reaction components and amplification process are similar to quantitative PCR, but some will contain the DNA target, whereas others will not. After amplification, the number of positive and negative reactions is determined and analyzed using the Poisson distribution. The recent development of micro- and nano-fluidic technologies makes digital PCR simpler and more practical (Morley, 2014). This technique is reproducible and can detect bacilli at concentrations as low as 1000 CFU/ml (Pholwat et al., 2013). With these advantages, digital PCR enables the earlier detection of new mutations emerging during treatment that could require a therapy change.

#### LATE-PCR With Lights-On/Lights-Off Probes

PCR-based detection of variant sequences is often carried out using probes labeled with different fluorescent dyes. However, the number of fluorescent dyes and the analysis capacity of a PCR machine are often limited to 4–6 colors. To overcome this problem, linear-after-the-exponential PCR (LATE-PCR), an asymmetric PCR method with sets of Lights-On/Lights-Off probes, has been developed to analyze nucleotide substitutions (Rice et al., 2012). In each probe pair, the Lights-Off probe is labeled with a quencher moiety and the Lights-On probe with a fluorescent molecule. The Tm of the probes are at least 5°C lower than that of the primers. After amplification, the number of single-stranded DNA molecules is much higher than that of double-stranded DNA molecules, due to the asymmetric PCR. The use of low temperature at the end of the process enables the hybridization of single-stranded DNA to the probes with lower Tm. After both probes are bound to their target sequence, the quencher of the Lights-Off probe extinguishes the fluorescence signal. Although each probe pair hybridizes only to a short part of the target, each fluorescent signal can be integrated to analyze sequences of several hundred nucleotides in length. The signal of the whole set of probes during the melting curve analysis can be used to create an annealing curve that shows the time-dependent fluorescence contour. In the case of mutations in the target sequence, the Tm of the hybridization changes, leading to a shift in the annealing curve. This new analytical technology is very sensitive for the detection of each single nucleotide change in a sequence of hundreds base pairs even with the use of a single fluorescent dye. The use of different color probes allows detecting various target genes in one single tube. However, the analysis of many different targets with multiple fluorescent dyes requires a powerful system. Besides, the use of one pair of modified probes for each mutation is expensive.

Hain Lifescience has applied this technology to develop FluoroType MTBDR VER1.0 for the simultaneously detection of the MTB complex and MDR-TB by targeting *rpo*B, *kat*G, and *inh*A in one single tube^11^. The kit includes probe pairs to target a set of known mutations in these genes (T508A, S509T, E510H, L511P, S512K, Q513L, Q513P, Q513R, D516A, D516F, D516V, D516Y, N518I, S522L, S522Q, H526C, H526D, H526G, H526L, H526N, H526P, H526Q, H526R, H526S, H526Y, R529K, S531F, S531L, S531L, S531Q, S531W, L533E, L533P in *rpo*B; S315T1, S315T2, S315N, S315R in *kat*G; and G-17T, A-16G, C-15T, G-9A, T-8A, T-8C, and T-8G in *inh*A), and can also detect unknown mutations, but not identify the exact type of mutation (Hillemann et al., 2018). Compared with GenoType MTBDRplus, FluoroType MTBDR is characterized by shorter hands-on time, faster results (within 3 h), no DNA contamination, and automatic result interpretation. The sensitivity and specificity are comparable to those of Genotype MTBDR*plus* and Xpert MTB/RIF for the detection of RIF resistance (Hillemann et al., 2018). However, they are lower than those of GenoType MTBDR*plus* for the detection of INH resistance-associated mutations in *kat*G and/or *inh*A. Hain Lifescience stated that the test can be used with decontaminated sputum and cultured samples^12^. More evaluation studies in different TB regions are needed before its implementation at TB point-of-care sites. WHO will start to evaluate the use of FluoroType MTBDR in the period 2018–2019 (WHO, 2017c).

### Sequencing

Sequencing is the best technology to rapidly analyze the genotype of an organism. Beside targeted gene sequencing (TGS), the development of Next Generation Sequencing (NGS) has been a major breakthrough in molecular biology because it can rapidly provide whole genome data in a single run (Phelan et al., 2016; Dheda et al., 2017; Manson et al., 2017). This allows species identification, screening of all (known and new) mutations (synonymous and non-synonymous mutations, insertions and deletions) in a sample, detecting drug resistance, and predicting the organism evolution. NGS-based kits for targeted sequencing have appeared on the market for DR-TB screening. For instance, Life technology has developed a novel protocol for rapid (2 days) full-length *Mycobacterium tuberculosis* gene analysis to detect first- and second-line drug resistance using the Ion Torrent Personal Genome Machine (PGM) (Daum et al., 2012). Eight genes (*rpo*B, *kat*G, and *inh*A, *pnc*A, *gyr*A, *eis*, *emb*B, and *rps*L) are amplified (PCR amplification of full-length genes, not of full genomes like for WGS) and sequenced. The AmpliSeq for Illumina TB Research Panel (Illumina) targets the same genome regions (see text foot note 12). Besides, WGS is now only valuable for cultured strains due to its requirement in terms of quantity and quality of DNA (WHO, 2018). Although some studies have performed WGS for direct sputum samples but the results were variable with a high level of human genome contamination. Currently, numbers of commercially novel NGS platforms such as Oxford Nanopore MinION (Oxford Nanopore Sequencing Technology, Oxford, United Kingdom) and PacBio RSII (Pacific Biosciences) are now available with advantage of short run time and long read. However, the massive output and high error rate are still main issues for their application (WHO, 2018). The Oxford Nanopore MinION, which is a small benchtop device that can plug directly into a laptop via a USB port cable, could generate 10–20 GB of data output per sample that requires a high speed computer and large storage space adequate for data analysis. In addition, the error rate is still high, up to 20–35%, but is expected to be improved as the MinION and its associated base-calling software continue to be developed (Senol Cali et al., 2018). Though, outstanding advantages of WGS including higher genome coverage, providing epidemiological information and understanding of new resistance mechanisms for both current and new drugs bring precious information to research and treatment of disease. Therefore, the use of WGS as a DR-TB detection tool could be potentially used in clinical settings in the future.

The large-scale application of sequencing, especially in middle- and low-income countries is still difficult for the following reasons: (i) robust software and database tools need to be developed for the full exploitation of this technology in this specialized area of medicine; (ii) specialized personnel and bioinformatics facilities are required for the experiments, data acquisition and data analysis; (iii) the high cost of NGS platforms; (iv) need to determine whether new mutations confer anti-TB drug resistance; and (v) high amounts of high quality DNA are required for sequencing (Phelan et al., 2016; Dheda et al., 2017; Zignol et al., 2018). Moreover, the sample preparation procedure and DNA extraction method should be standardized for consistency (Zignol et al., 2018).

Nevertheless, the cost of sequencing is progressively decreasing and is already lower than that of phenotypic testing for first- and second-line drug resistance in most settings (Dheda et al., 2017; Zignol et al., 2018). The Foundation for Innovative Diagnostics (FIND) and their partners are developing a system designed to perform targeted amplicon sequencing directly from primary sputum samples (Dolinger et al., 2016). Several studies have reported that WGS can be successfully performed directly using sputum samples thanks to new strategies, such as targeted DNA enrichment (McNerney et al., 2017; Doyle et al., 2018). The procedure is progressively becoming simpler, affordable and rapid. NGS and TGS are promising tools for the surveillance of drug resistance and the WHO has published a technical guideline for use of NGS technology for the detection of mutations associated with drug resistance in MTB (WHO, 2018). Two consortiums of NGS experts, the ReSeqTB Consortium and CRyPTIC, have established huge repositories of genomic and phenotypic data accumulated from thousands of MTB strains worldwide. These consortiums provide platforms that allow automatic processing raw WGS data to comprehensive mutation lists and drug resistance panels for users that are convenient for non-expert bioinformatics^13^. Currently, these platforms are only available for surveillance and research use. Ultimately, they will be interoperable and transferred to the WHO to support global DR-TB surveillance in the short term and clinical diagnosis of DR-TB in the long term (WHO, 2018).

### DNA Microarray

Apart from sequencing, the DNA microarray technology displays the highest capacity of multiple target detection with thousands of sequences in one reaction. In many studies, this technology has been used for detecting mutations associated with drug resistance in MTB. After DNA extraction, the targets are amplified and labeled with fluorescent dyes during the PCR step (Noyer et al., 2015). Then, the labeled amplicons are complementarily hybridized to the probes immobilized on the array to form double-stranded DNA. Non-specific targets and non-specific binding are eliminated in the post-hybridization washing steps. The hybridization signal intensity is detected by a scanner at the appropriate wavelength. Different kinds of fluorescent dyes are used, such as Cy3 (Naiser et al., 2008) and Cy5 (Noyer et al., 2015). Often, very short targets are amplified (from 60 to 300 bp) to optimize the hybridization step. The probe length varies between 10 and 40 bp (Naiser et al., 2008; Yao et al., 2010; Noyer et al., 2015). Two-round PCR amplification (Yao et al., 2010) or multiplex PCR (classical or asymmetric) (Shimizu et al., 2008; Zimenkov et al., 2013; Linger et al., 2014) are generally used to obtain sufficient amplicons for hybridization. However, the hybridization procedure varies between studies [55°C for 2 h (Yao et al., 2010), 42°C for 60 min (Shimizu et al., 2008), or 37°C for 10–16 h (Zimenkov et al., 2013)]. A threshold signal-to-noise ratio (often higher than 3) is defined to avoid false results (Shimizu et al., 2008; Yao et al., 2010; Zimenkov et al., 2013; Linger et al., 2014).

To simplify the numerous steps of the microarray-based workflow, Akonni developed the TruArray MDR-TB Assay that uses a microfluidic chamber to integrate almost all steps (amplification, hybridization and target detection) in one platform^14^ (Linger et al., 2014; Maltseva et al., n.d.), and to limit the risk of cross-contamination. The assay covers up to 30 *rpo*B mutations, 6 *kat*G and *inh*A mutations, 1 *emb*B mutation, and 2 *rps*L mutations. In addition, it targets the IS6110 and IS1245 markers for the detection of the MTB complex and *M. avium* complex, respectively. The results are available in few hours. VereMTB^TM^ (Veredus Laboratories, Singapore) is another kit based on a multiplexed PCR-microarray-based method and microfluidic chamber to detect MDR-TB, MTB and nine other *Mycobacterium* species^15^. Interestingly, Zimenkov et al. (2016) recently developed a low-density hydrogel microarray (TB-TEST) that enables to detect simultaneously first- and second-line drug resistances in one run. This microarray was developed from two previous biochips (TB-Biochip, one for MDR and one for XDR) with an additional of targeting *emb*B gene and 12 probes for identification of the main lineages circulating in Russia. By using two separate asymmetrical PCRs and universal adapters, this array covers nine genes including *rpo*B, *kat*G, *inh*A, *ahp*C, *gyr*A, *gyr*B, *rrs*, *eis*, and *emb*B genes and six SNPs for lineage identification. The turn-around time is about 19 h (3 h for PCR and 16 h for hybridization), that is quite long compared to other molecular-based methods but detecting both MDR-TB and XDR-TB is the greatest advantage of this microarray. The sensitivity and the specificity of TB-TEST are identical between clinical samples and clinical isolates. This microarray has high potential to apply directly in clinical samples.

Currently, no microarray-based assay has been endorsed by WHO for the detection of DR-TB (WHO, 2017c). Most tests are still in development. The GeneChip MDR Kit (CapitalBio, China) is the only microarray-based assay on the market that has been evaluated in the framework of the NHFPC-Bill & Melinda Gates Foundation tuberculosis project, but not by WHO (CapitalBio, n.d.; WHO, 2017c). It is considered to be an accurate and feasible tool with high sensitivity and specificity for the direct detection of RIF and INH resistance in clinical samples in China (Zhang Z. et al., 2012). In a study using spinal tuberculosis samples, this test showed a sensitivity and specificity of 88.9 and 90.7%, respectively, for RIF resistance detection, and of 80 and 91%, respectively, for INH resistance screening (Zhang Z. et al., 2012). The GeneChip MDR Kit has been widely implemented in different provinces of China^16^.

Overall, DNA microarray-based tests show high specificity and sensitivity for the detection of mutations associated with anti-TB drug resistance in clinical isolates: 100% sensitivity and specificity for INH and RIF detection (Yao et al., 2010); and 90 and 95.7% sensitivity for the detection of resistance to FQ, and SLIDs, respectively, with a specificity of 90.9% for FQ resistance and 90.2% for SLIDs (Zimenkov et al., 2016). The lowest sensitivity (89.9%) and specificity (57%) is for EMB resistance. For the detection of resistance to RIF, INH, STR, EMB and KAN in sputum samples from patients with TB, specificity varied from 60 to 95%, and sensitivity was higher than 90% (Shimizu et al., 2008). The microarray detection limit varies among platforms and studies. The GeneChip MDR Kit (CapitalBio) could detect at as low as 25 genome copies (Guo et al., 2009; Zhang Z. et al., 2012). The microarray developed by Linger et al. has an analytical sensitivity of 110 genome copies per assay (Linger et al., 2014). Zimenkov et al. (2013) tested the biochip using various clinical specimens, such as sputum and bronchoalveolar lavage samples, and found that its diagnostic sensitivity varies according to the smear grade, from 67% for smear “1+” to 100% for smear “3+.”

DNA microarray could become an effective method for the rapid screening of MTB resistance. However, additional improvements, such as dedicated software to analyze and interpret the fluorescent signal intensity, are necessary to make it accessible to all TB point-of-care sites and promote its application in the clinical routine.

## Challenges for the Development of Molecular Markers for the Detection of DR-TB

Many molecular tools for DR-TB diagnosis have been developed and are employed worldwide. However, there are still many challenges and issues that need to be solved to provide efficient and affordable point-of-care diagnostic tests.

**Figure 1:**
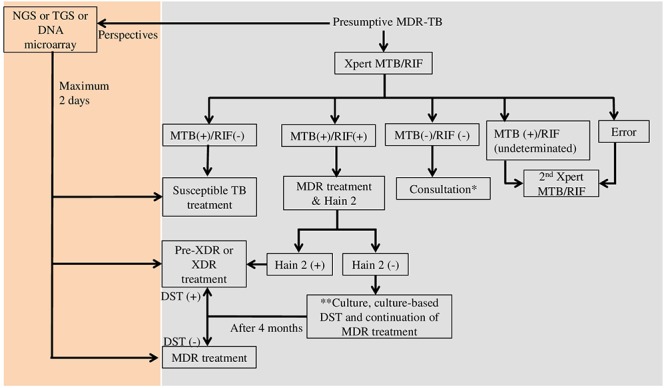
Diagnostic flowchart for presumptive multi-drug resistant tuberculosis in Vietnam (Vietnam’s Ministry of Health) and perspectives. MTB, *Mycobacterium tuberculosis*; RIF, rifampicin; Hain 2, GenoType MTBDR*sl* (Hain Lifescience, Germany); MDR, multi-drug resistance; XDR, extensively drug resistance; NGS, next generation sequencing; TGS, targeted gene sequencing; DST, drug susceptibility testing; ^∗^If the result of consultation is non-TB, non-tuberculosis mycobacteria identification by culture is recommended; ^∗∗^Culture and culture DST are performed when the patient is still suspected of having second-line drug resistant TB.

Technically, most of the tests only focus on one or few genes, hotspot genomic regions, or frequent mutations (Supplementary Table S1). Therefore, one sample should use several tests to get the complete drug-resistance genotype and to choose the most appropriate treatment for each patient. In Vietnam, a middle-income country, presumptive MDR-TB cases such as TB patients with treatment failure or HIV /TB co-infected patients are tested with Xpert MTB/RIF (Vietnam’s Ministry of Health, 2018) (see Figure 1). Many steps are necessary from diagnosis to treatment, especially for the diagnosis of XDR-TB cases. The total diagnostic time might be extended up to 4 months. The application of molecular tests such as NGS, TGS, or DNA microarray would reduce the diagnostic time to only 2 days. Moreover, the mutation frequency and distribution vary among populations, and many mutations linked to DR-TB are outside the regions targeted by commercial kits. Consequently, the sensitivity and specificity of a test can vary in different populations. An ideal test should cover all mutations worldwide to provide high sensitivity and specificity. The number of fluorophores, target size, and the complexity of investigating multiple targets are the main limitations that affect the test design and efficiency. DNA microarray could overcome the multiple target limitation because it allows querying thousands of sequences with only one fluorophore in one assay. Several studies demonstrated that DNA microarray can detect low numbers of DR-TB bacilli with high sensitivity and specificity in clinical specimens (Shimizu et al., 2008; Guo et al., 2009; Zimenkov et al., 2013). Several microarray-based devices are currently in development or under evaluation to be used in clinical settings (WHO, 2017c). However, a simpler platform and an easier assay/analysis are necessary to implement DNA microarray as a routine test.

Drug resistance emerges quickly after the introduction of new anti-TB drugs (Bañuls et al., 2018). To develop accurate genetic-based diagnostic tests for DR-TB, the first crucial step is to understand the molecular mechanisms of resistance. For example, a recent review alerted about resistance of MTB to bedaquiline, a new potential drug for the treatment of DR-TB, and its cross-resistance with clofazimine (Nguyen T.V.A. et al., 2017). The mechanism of resistance was linked to several genes: *atp*E, *Rv0678*, and *pep*Q. However, it is only a non-exhaustive list because a standardized DST for bedaquiline does not exist yet, and not all resistance genotypes are known. Due to MTB high capacity of drug resistance acquisition, the development of a standardized DST should be started simultaneously with the evaluation of a new drug in clinical trials. Thereby, DST would be available when the new drug will be introduced in treatment regimens. However, it is a long and difficult path. For instance, although pyrazinamide has been used for many years, the standardized DST for this drug is still “work in progress” due to its current not satisfactory accuracy and reproducibility (Chedore et al., 2010; Huy et al., 2017; Zignol et al., 2018). Thus, the development of quick molecular tests for drug resistance is still limited by the lack of highly accurate standard phenotypic tests, as reference.

Harsh climatic and environmental conditions are another big issue for the development of a molecular test. For instance, the Xpert machine is sensitive to dust and temperature (Walzl et al., 2018). In high TB-burden countries, such as India and Vietnam, the harsh climate conditions (intense heat, high humidity, and pollution) require setting up air-conditioned laboratories and regular maintenance check-ups to ensure the shelf life of the machine and the test accuracy. This kind of working environment might cost more than the machine itself. Although the Xpert test is rapid and easy to use, such difficulties hamper its implementation in low- or middle-income countries.

Besides these issues, commercial pressure might limit the screening of optimal biomarkers (Walzl et al., 2018). The numerous steps from the design/development to the scaled-up manufacture and marketing might take more than 10 years and cost more than $100 million. Thus, although many academic and private-sector research groups have been working on the development of novel diagnostic tools, only few tools were endorsed by WHO because most of them do not meet the required performance standards.

## Conclusion

The progressive increase of DR-TB cases emphasizes the vital need for accurate and rapid diagnostic tools for their detection. The main current molecular techniques are LPA, real-time PCR, DNA microarray and sequencing. Cost, shelf life, sample throughput and accuracy are key factors for the development and application of such tests/systems. Other factors should also be considered, such as target capacity (i.e., how many drug resistance mutations can be detected) and the personnel skills required for running the test. Furthermore, to have a real impact, molecular diagnostic tests should be affordable for resource-poor countries, where TB and DR-TB are major problems. The turnaround time should be as short as possible to quickly prescribe the adapted treatment to the patients. As DR-TB is associated with mutations in different genes, an ideal test should simultaneously detect many mutations in one reaction. A simple testing procedure will increase the test accessibility at different laboratory levels.

## Author Contributions

All authors contributed to the review, manuscript writing, critical review of the manuscript, and approved the final manuscript.

## Conflict of Interest Statement

The authors declare that the research was conducted in the absence of any commercial or financial relationships that could be construed as a potential conflict of interest.

## Footnotes

1 https://www.bd.com/en-uk/products/diagnostics-systems/identification-and-susceptibility-systems/bactec-mgit-

2 http://www.hain-lifescience.de/en/technologies/dnastrip.html

3 https://www.aid-diagnostika.com/en/kits/molecular-genetic-assay/infections-diseases/antibiotic-resistences/tb-isoniazid-rifampicin/

4 https://www.hain-lifescience.de/en/products/microbiology/mycobacteria/tuberculosis/genotype-mtbdrplus.html

5 http://www.hain-lifescience.de/en/products/microbiology/mycobacteria/tuberculosis/genotype-mtbdrsl.html

6 http://www.bio-rad.com/fr-fr/applications-technologies/what-real-time-pcr-qpcr

7 http://www.who.int/tb/features_archive/Xpert-Ultra/en/

8 https://www.tbfacts.org/genexpert/

9 https://www.genedrive.com/documents/Clinical-performance-evaluation-Genedrive-MTB_RIF-assay-India-July-2016.pdf

10 http://www.seegene.com/neo/en/products/tuberculosis/tuberculosis_list.php

11 http://www.hain-lifescience.de/en/products/microbiology/mycobacteria/tuberculosis/fluorotype-mtbdr.html

12 https://www.illumina.com/products/by-brand/ampliseq/community-panels/tb.html

13 https://platform.reseqtb.org/

14 https://akonni.com/products/trudiagnosis/truarray-mdr-tb-assay/

15 https://vereduslabs.com/products/clinical/veremtb/

16 http://en.thholding.com.cn/2016-08/22/c_56177.htm
