# Supplementary material for: Molecular Diagnosis of Drug-Resistant Tuberculosis; A Literature Review
Source: Front Microbiol. 2019 Apr 16;10:794. doi: 10.3389/fmicb.2019.00794 (PMC6477542; doi:10.3389/fmicb.2019.00794)
Supplement: Supplementary file 1 [file Data_Sheet_1.PDF]

Supplementary Material

Molecular Diagnosis of Drug-resistant Tuberculosis; a Literature Review

Thi Ngoc Anh Nguyen*, Véronique Anton-Leberre, Anne-Laure Bañuls[1], Thi Van Anh Nguyen[1],

Correspondence: Thi Ngoc Anh Nguyen*

Email: anhnguyen.011188@gmail.com

[1]These authors contributed equally to this work

Table S1: Molecular diagnostic tests for the detection of drug resistance in MTB

**Test characteristic**	**Hain LifeScience GenoType MTBDR*plus* VER2.0**[α]	**Hain LifeScience GenoType MTBDR*sl* VER2.0**[α]	**Nipro NTM/MDR-TB strip**[α]	**Nipro INH strip**	**Nipro PZA strip**	**Nipro FQ strip**
Target	*rpo*B 505-533; *kat*G 315; *inh*A -8, -15, -16[a]	*gyr*A 90-95; *gyrB* 485-538; *rrs*1401& 1484[a]; *eis* -14[a]	*rpo*B 484-534; *kat*G 294-315; *inh*A -17 to -3[b]; 6-11	*inh*A -17 to -3[b] 6-11; *fab*G1 194-206. *fur*A 6-16; *kat*G 45-712	*pncA* -17 to 4[c] 1-185	*gyrA* 88-97
**Intended use**	RIF and INH resistance	FQ and INJ resistance	RIF and INH resistance; 4 *Mycobacterium* species	INH resistance	PZA resistance	FQ resistance
**Principle of the test**	Line probe assay	Line probe assay	Line probe assay	Line probe assay	Line probe assay	Line probe assay
**Turn-around time**	5h	5h	7h	7h	7h	7h
**Sample throughput**	Smear-positive specimens and cultured isolates	Smear-positive specimens and cultured isolates	Smear-positive specimens and cultured isolates	Smear-positive specimens and cultured isolates	Smear-positive specimens and cultured isolates	Smear-positive specimens and cultured isolates
**Limits of detection**	Smear-positive specimens*	Smear-positive specimens*	Smear-positive specimens*	Smear-positive specimens*	Smear-positive specimens*	Smear-positive specimens*
**Drawbacks**	Limited number of targets; high rate of uninterpretable results	Limited number of targets; high rate of uninterpretable results	Limited number of targets	Limited number of targets	Limited number of targets	Limited number of targets

Test characteristic	Innogenetics INNO-LIPA Rif TB	Diagnostika GmbH AID TB	Cepheid GeneXpert MTB/RIF[α]	Cepheid GeneXpert MTB/RIF ultra[α]	Seegene Anyplex II MTB/MDR	Seegene Anyplex II MTB/XDR
**Target**	*rpo*B 509-534	*rpo*B513-533; *kat*G 315; *inh*A-8, -15, -16[a]; *rps*L43 & 88; *rrs*1401, 1402 &1484[a]; *gyr*A90, 91, 94; *emb*B 306	*rpo*B 507-533	*rpo*B 507-533	*rpo*B 511, 513, 516, Deletion 513-516, 522 ,526, 531	*gyr*A 90, 91, 94; *rrs*1401,1402, 1484; *eis* –37, –14, –10
**Intended use**	RIF resistance	RIF, INH, Aminoglycoside, FQ and EMB resistance	MTB and RIF resistance (MDR-TB)	MTB and RIF resistance (MDR-TB)	MTB and MDR-TB	MTB and XDR-TB
**Principle of the test**	Line probe assay	Line probe assay	Nested real-time PCR with molecular beacon probes	Nested real-time PCR with molecular beacon probes	Multiplex real-time PCR with the DPO[TM] and TOCE[TM] technology	Multiplex real-time PCR with the DPO[TM] and TOCE[TM] technology
**Turn-around time**	6h	4h	2h	2h	3.5h	3.5h
**Sample throughput**	Bacterial cultures on solid medium	Cultured isolates and smear-positive clinical specimens	Sputum specimens	Sputum samples and extra-pulmonary specimens	Cultured isolates and clinical specimens	Cultured isolates and clinical specimens
**Limits of detection**	Cultured isolates*	> smear 1+	131 bacilli per ml of sputum	16 bacilli per ml of sputum	Negative AFB smears*	Negative AFB smears*
**Drawbacks**	One targeted gene	Limited number of targets	Limited number of targets; False positive results due to silent mutations	Limited number of targets; Low specificity for MTB detection	Limited number of targets	Limited number of targets

Test characterist-ic	Epistem Genedrive MTB/RIF ID Kit	Hain LifeScience FluoroType MTBDR VER1.0[β]	TB-TEST (Russia)	Capital Bio TB drug resistance array	AutoGenomics INFINITI System MDR-TB BioFilm Chip Microarray	Akoni TruArray MDR-TB
**Target**	*rpo*B 516-531	*rpo*B 508-533 ; *kat*G 315 ; *inh*A -8, -9, -15, -16, -17[a]	*rpo*B 507-533*; kat*G *315-328; inh*A (-24)-(-8; *ahp*C (-12)-(-6)*; gyr*A 70-95*; gyr*B *485-540; rrs* 1401-1484*; eis* (-37)-(-10); *emb*B 296-328; *IS6110; Rv0557; Rv1811; Rv2629; oxcA* and *Rv0129c*	*rpo*B 511-533; *kat*G 315; *inh*A -15[a]	*rpo*B511-533; *kat*G 315; *inh*A-15[a]; *pnc*A 57 & 100	*rpo*B 507, 510-513, 515, 516, 522, 524, 526, 531, 533; *kat*G 315; *inh*A -8, -15, -17[a]; *emb*B 306; *rps*L 43 & 88; IS6110&IS1245
**Intended use**	MTB and RIF resistance	RIF and INH resistance	MDR-TB and XDR-TB, MTB lineage	MTB RIF and INH resistance	RIF, INH and PZA resistance	RIF, INH, STR and EMB resistance; MTB complex and *M. avium* complex
**Principle of the test**	Multiplex real-time PCR	LATE PCR with Lights-On/Lights-Off probes	Multiplex PCR and low-density hydrogen microarray	PCR and microarray	PCR and microarray	Multiplex PCR and gel element microarray
**Turn-around time**	75min	3h	19h	5.8h	NA	24 samples/8h
**Sample throughput**	Clinical sputum samples	Decontaminated sputum and cultured samples	Clinical samples and cultured isolates	Clinical specimens and cultured samples	Decontaminated cell lysates	Cultured isolates; smear-positive clinical specimens
**Limits of detection**	30 cfu/ml	Decontaminated sputum and cultured samples*	200 copies/reaction	≥ 25 copies (Guo et al., 2009; Zhang et al., 2012)	25-50ng genomic DNA (Mahant, et al. 2003)	110 copies /assay
**Drawbacks**	Limited number of targets;	Limited number of targets	Manual and laborious	Semi-automatic, Requires technical skills	Requires high sample throughput	NA

[α]Tests endorsed by WHO;[β]WHO scheduled for evaluation in 2018/2019; *No specific number; [a]Nucleotide position; [b]Nucleotide position relative to the start codon of *fab*G; [c]Nucleotide position relative to the start codon of *pnc*A; NA: Not available information; DPO™: dual-priming oligonucleotides; TOCE™: tagging-oligonucleotide cleavage and extension; $: US dollar; h: hour; min: minutes; INH: isoniazid; RIF: rifampicin; STR: streptomycin; EMB: ethambutol; FQ: fluoroquinolone; PZA: pyrazinamide; INJ: second-line injectable drugs; MDR-TB: multi-drug resistant tuberculosis; XDR-TB: extensively drug resistant tuberculosis;

References

Eddabra, R., and Benhassou, H. A. (2018). Rapid molecular assays for detection of tuberculosis, 1–12. Pneumonia.

Guo, Y., Zhou, Y., Wang, C., Zhu, L., Wang, S., Li, Q., et al. (2009). Rapid, accurate determination of multidrug resistance in *M. tuberculosis* isolates and sputum using a biochip system. *International Journal of Tuberculosis and Lung Disease*, *13*(7), 914–920.

Pang, Y., Li, Q., Ou, X., Sohn, H., Zhang, Z., Li, J., et al. (2013). Cost-Effectiveness Comparison of Genechip and Conventional Drug Susceptibility Test for Detecting Multidrug-Resistant Tuberculosis in China. *PLoS ONE*, *8*(7), 6–11. doi: 10.1371/journal.pone.0069267

Vijay Mahant, Fareed Kureshy, Ram Vairavan, and G. H. (2003). THE INFINITI™ SYSTEM- AN AUTOMATED MULTIPLEXING MICROARRAY PLATFORM FOR CLINICAL LABORATORIES. *Microarray Methods and Applications-Nuts & Bolts* (pp. 325–338).

Zhang, Z., Li, L., Luo, F., Cheng, P., Wu, F., Wu, Z., et al. (2012). Rapid and accurate detection of RMP- and INH- resistant *Mycobacterium tuberculosis* in spinal tuberculosis specimens by CapitalBio™ DNA microarray: a prospective validation study. *BMC infectious diseases*, *12*, 303. BioMed Central. doi: 10.1186/1471-2334-12-303
